# Italian Coma Recovery Scale for Pediatrics (CRS-P): Preliminary Validation in a Sample of Young Children with Typical Development

**DOI:** 10.3390/brainsci15020118

**Published:** 2025-01-26

**Authors:** Katia Colombo, Claudia Corti, Chiara Porro, Claudia Fedeli, Monica Beschi, Cristina Reverberi, Sandra Strazzer

**Affiliations:** 1 Acquired Brain Injury Unit, Scientific Institute, IRCCS E. Medea, Bosisio Parini, 23842 Lecco, Italy; katia.colombo@lanostrafamiglia.it (K.C.); chiara.porro@lanostrafamiglia.it (C.P.); claudia.fedeli@lanostrafamiglia.it (C.F.); sandra.strazzer@lanostrafamiglia.it (S.S.); 2 Don Serafino Ronchi Rehabilitation Center, Montichiari, 25018 Brescia, Italy; monicabeschilogopedista@gmail.com; 3 Department of Health Professions, AUSL-IRCCS Reggio Emilia, 42122 Reggio Emilia, Italy; cristina.reverberi@unimore.it

**Keywords:** disorders of consciousness, vegetative state, minimally conscious state, children, pediatric, assessment, neurobehavioral

## Abstract

Background/Objectives: Guidelines for the diagnosis of children with disorder of consciousness (DoC) in pediatric age have not been defined yet. Assessment tools designed for adults have generally not been standardized for pediatrics, which could lead to misdiagnosis due to the limited behavioral repertoire of children. This study aims at examining the basic psychometric properties of the Italian Coma Recovery Scale for Pediatrics (CRS-P) in typically developing children. Methods: A total of 64 typically developing children aged 3 months to 5:7 years were administered the CRS-P. Performance was examined across the age range, and for the two behaviors indicating emergence to a conscious state, namely functional object use (FOU) and functional communication (FC). Results: Inter-rater reliability ranged from 0.95 to 1 for subscale and total scores. All children aged ≥34 months scored at the CRS-P ceiling. All children ≥ 14 months met the criteria for FOU and all children ≥ 34 months met those for FC. Children as early as 3 months of age displayed behaviors discriminating between vegetative state (VS) and minimally conscious state (MCS) in the Visual and Motor subscales. Language-based behaviors of MCS in other subscales were consistently displayed by older children. Conclusions: Typically developing children met the criteria for all items of the Italian CRS-P by 34 months, which suggests caution in adopting the scale at a younger age. However, the features of the distinct stages of DoC could be captured earlier, based on the various subscales. Modifications should be made to some items to improve diagnostic accuracy.

## 1. Introduction

A disorder of consciousness (DoC) is a state of prolonged altered consciousness due to an acquired brain injury, leading to alterations of arousal, awareness, and responsiveness. Standardized criteria for defining the distinct stages of a DoC were set in the early 2000s [1,2]. It is commonly shared that DoC classification includes coma, vegetative state/unresponsive wakefulness syndrome (UWS), minimally conscious state (MCS), covert cognition, and locked-in-state [3,4,5,6]. MCS was further subcategorized into two sub-groups according to the presence (MCS+) or absence (MCS-) of language [4,7].

Recent studies have highlighted the lack of research on pediatric DoC, especially in children under 5 years of age, with even more limited data on behavioral characteristics for children under one year of age or with significant motor impairments [8,9]. Further, the limited evidence on the topic is mainly provided by studies with methodological shortfalls, such as cases, series, or observational studies [10]; the few large cohort studies including children often lacked detailed demographic and clinical information [10]. A survey conducted in 2019 [11] and recent guidelines [12] have highlighted a lack of appropriate tools and standards for pediatrics and the paucity of guidelines and recommendations for admission to acute care and rehabilitation centers.

One of the major challenges in the management of pediatric DoC is the limited behavioral repertoire of infants and very young children, due to the immaturity of the nervous system, along with the fluctuation of responses and the rapid development of cognitive and behavioral skills [8,13,14]. In addition, the relation between outcome and age at injury and the difficulty in disentangling the evolution of the conscious state from the physiological grow curve make the behavioral assessment of children harder than in adults [15,16,17,18,19]. The adoption of neurobehavioral assessment tools designed and validated on adult patients with very young children could lead to inaccurate classification due to poor language development and sensorimotor limitations [8]. However, such an assessment is of fundamental importance to predict future outcomes and plan rehabilitation.

For this reason, in recent years, some researchers and clinicians have made an increasing effort in summarizing the evidence on DoC and evaluating the applicability of tools originally developed for adults in young patients [8,9,10,11,20,21,22,23,24,25]. The Coma Recovery Scale-Revised (CRS-R) [26] is traditionally considered the gold standard of bedside assessments for patients with DoC [12]. Even though this scale was originally developed for adults, it is also recommended for the assessment of children in the absence of other validated tools for this age group [12]. However, especially in younger children, CRS-R is difficult to apply [25].

Over the past two decades, our research team has dedicated significant effort to evaluating the possibility of adopting tools originally developed for adults to assess DoC in children in our country. In a prospective observational study published in 2023 [25], the CRS-R [26] was experimentally applied to describe the level of consciousness in children and adolescents. This study [25] also aimed to explore the agreement between CRS-R [26] and another standard index scale used with adults, namely the Rappaport Coma Near Coma Scale (CNCS) [27]. A moderate agreement between the two scales was found, especially in the visual and auditory domains. However, the CRS-R [26] demonstrated high motor demands, potentially being too challenging for children emerging from MCS who present with significant motor impairment. Additionally, CRS-R [26] was found to be applicable for tracking changes in DoC in children as young as 5 years of age, but it was inapplicable with younger children [25]. Our group has also previously tested the Rappaport CNCS [27] in a sample of children [22]. Results, reported in a study published in 2020 [22], indicated that, when used in combination with the Levels of Cognitive Function Assessment Scale (LOCFAS) [28,29], it could reduce the rate of false negatives and better detect signs of arousal and awareness. LOCFAS [28,29], whose usability in children was tested by our group in 2008 [24], was later found to enable practitioners to detect minor changes and value minimal improvements in patients appearing as stable [13]. However, the scale was shown to be inapplicable to children younger than 4 years of age in practice [22]. Therefore, despite these efforts, there remains a significant need to develop and test tools that can allow for the early and accurate detection of emergence from disorder of consciousness in children, while also capturing the behavioral characteristics of very young patients.

A pediatric version of the CRS-R for children under 5 years of age (Coma Recovery Scale for Pediatrics; CRS-P) [20], consisting of the same six subscales of the original CRS-R [26], was adapted by Slomine and collaborators in 2019 for English-speaking countries [20]. The scale was able to differentiate between responses compatible with arousal in UWS and emergence from MCS (eMCS). Some promising psychometric properties were also collected, even though some modifications are still required to correctly capture all behavioral repertoires of young patients [20]. CRS-P was found to be appropriate for use in children as young as 12 months of age in the preliminary validation study carried out in the United States [20]; based on these encouraging findings, we aimed to test the tool for use in the Italian context as well.

In the present study, the Italian version of CRS-P, whose translation and adaptation process for the country was described in detail in a recent paper [30], was administered to children with typical development in order to assess the basic psychometric properties of the scale for the Italian context. In accordance with the preliminary validation of the original scale [20], an examination of children’s behavioral responses to CRS-P items at different age levels was conducted, and the age at which the ceiling for each item was reached was investigated.

This study may contribute to the development of objective clinical evaluation tools for children with DoC, also considering the limited existing national guidelines for diagnostic and prognostic procedures for this population [11]. Examining the performance of typically developing children is the preliminary step in assessing the clinical application of CRS-P in children with brain injury in the same age group. Indeed, it helps identify the expected behavior of typically developing children in various cognitive and motor domains during early development. This information is crucial for determining the minimum age at which a specific ability is expected to be displayed. This, in turn, allows detecting deviations in the growth curve of children with DoC. Unlike adults, in pediatrics, there is the need to acknowledge whether a behavior occurs or not at a given age in typically developing children in order to then evaluate whether it may be displayed also by children with DoC. This may limit the possibility to attribute to the DoC the absence of a behavior that does not normally occur due to age reasons. Finally, comparisons in the achievement of developmental milestones between healthy children and those with DoC can help identify high-priority rehabilitation goals for the latter.

## 2. Materials and Methods

### 2.1. Measure

The Italian version of CRS-P [30] was administered to a sample of 64 healthy children. The original CRS-P developed by Slomine and collaborators [20] constitutes the adaptation for use in pediatrics of the CRS-R [26], which was designed for adults. The main modifications to the tool for use with children included the use of child-friendly objects and toys and language adaptation to facilitate child comprehension and interaction [20].

The Italian CRS-P [30] is an adaptation and translation for the Italian context of the original CRS-P in English [20]. As clearly detailed in the paper on the Italian translation of the scale [30], the methodological approach used for the adaptation process allowed for maintaining conceptual, semantic, and content correspondence with the original version. The Italian CRS-P [30] includes 29 items hierarchically organized into the following 6 subscales: Auditory (score range: 0–4), Visual (score range: 0–5), Motor (score range: 0–6), Oromotor/Verbal (scores range: 0–3), Communication (score range: 0–2), and Arousal (score range: 0–3). The total score ranges from 0 to 23 (the higher the score, the better the performance), and is obtained by summing up each item score. Every single subscale score allows obtaining the specific DoC classification (VS, MCS, eMCS). Across CRS-P administration, each patient is tested on the ability to correctly answer to stimuli presented in a standardized manner. The lowest items rely on reflexive reactions, while the highest rely on cognitive-mediated activities.

### 2.2. Participants

The inclusion criteria to partake in the study were the following:

- Birth at full term (at least 38 weeks gestation).
- Absence of a diagnosis of developmental or neurocognitive disorders.
- Italian as native language.

The exclusion criteria were as follows:

- Delays in exhibiting developmental milestones, as reported by caregivers.
- Being in foster care.

A total of 64 Caucasian typically developing children (51.6% males) aged 3 months to 5:7 years (67 months) (mean = 18.69 months; median = 10.04 months; interquartile range (IQR) = 44.60 months) at the time of examination participated in the study. The maternal education level corresponded to a bachelor’s degree in 83% of cases. The distribution of children by age group was the following: 10 children were included in the 0–<6 month group, 10 in the 6–<12 month group, 8 in the 12–<18 month group, 8 in the 18–<24 month group, 6 in the 2–<3 year group, 9 in the 3–<4 year group, and 7 in the 4–<5 year group.

### 2.3. Procedures

Two independent clinical psychologists with many years of experience in assessing and treating children with DoC performed the assessments through the Italian CRS-P. For 44 children, the assessment was performed in parallel by two psychologists to allow for the measurement of inter-rater reliability. For the remaining 20 children, the examination was conducted by a single clinician.

As for the original CRS-P [20], all items of each subscale were administered. The administration of each subscale was carried out starting with the item with the lowest score. All the possible modes of administration of each subscale were adopted, to favor the emergence of children’s full range of performance. In detail, for the Auditory subscale, the entire set of alternative commands was administered; for the Communication subscale, all situational orientation question sets were used. Items including potentially aversive stimuli (e.g., noxious stimulation) were administered at the end of the evaluation to maintain compliance and limit children’s distress. In addition, to facilitate children’s adjustments to the assessment setting, the presence of at least one parent in the assessment room was ensured; responses given to parental requests (each parent was previously instructed appropriately) were considered valid if the child did not respond to the psychologist first.

### 2.4. Data Analysis

To evaluate the inter-rater reliability of the CRS-P subscale scores, Lin’s concordance correlation coefficient between two evaluations performed by two clinical psychologists was calculated.

Demographic characteristics of participants and subscale scores by age groups were calculated by descriptive statistics. For age groups, the following increments were considered: 6-month increments from 0 months to <2 years and 1-year increments for 2, 3, 4, and 5-year-olds. Correlations among CRP-S scores and age were calculated by using Pearson correlation coefficient.

## 3. Results

### 3.1. Inter-Rater Reliability

Lin’s concordance correlation coefficient ranged from 0.95 to 1 for all subscale and total scores. Medians [ranges] and means (standard deviations—SDs) on the CRS-P subscale and total scores assigned by each psychologist and the related Lin’s concordance correlation coefficient are reported in Table 1.

### 3.2. Total and Subscale Scores by Age

Age-dependent descriptive statistics for the CRS-P total and subscale scores are reported in Table 2.

For the total score (corresponding to 23), all twenty-two 3–5:7 year olds and four of the six 2-year olds scored at the ceiling of the CRS-P. The two 2-year olds who did not reach the ceiling of the CRS-P did not obtain the highest score on the Communication and Arousal subscales.

Age was significantly correlated with total and subscale scores (total score, r = 0.88, *p* < 0.01; Auditory, r = 0.83, *p* < 0.01; Visual, r = 0.73, *p* < 0.01; Motor, r = 0.55, *p* < 0.01; Oromotor/Verbal, r = 0.82, *p* < 0.01; Communication, r = 0.89, *p* < 0.01; Arousal, r = 0.90, *p* < 0.01).

#### 3.2.1. Auditory Subscale

All 2-year-olds scored at the ceiling in the auditory subscale, having a score of 4. The youngest child who achieved the highest score in this subscale was 15 months old. All children showed auditory startle and by 13 months were all able to localize sounds. Moreover, 90% of very young children (<6 months) exhibited localization to sound.

#### 3.2.2. Visual Subscale

All children exhibited visual startle. By 2 years, all children exhibited object recognition, thus reaching the ceiling in this subscale. By 1 year, all children consistently displayed visual pursuit. All children by 1 year exhibited object localization (reaching). However, 5 of the total 14 children aged between 5 and 12 months spontaneously showed object localization (reaching; score = 4), but did not meet the criteria for the previous item, i.e., visual pursuit (score = 3). This was because they demonstrated spontaneous visual pursuit when an object was present, especially if it was child-friendly, but did not show such behavior when a mirror was used, as required by the original CRS-P [20] administration procedure. Moreover, 50% of very young children (<6 months) exhibited visual pursuit.

#### 3.2.3. Motor Subscale

No children displayed abnormal posturing, but all exhibited flexion withdrawal. Only a child aged 4 months displayed a response of localization to noxious stimulation. Additionally, 40% of very young children (<6 months) exhibited automatic motor response. By 18 months, all children reached the subscale ceiling corresponding to functional object use (FOU), which constitutes one of the two criteria signaling the emergence from DoC. The 18 children who did not display FOU were all <14 months.

#### 3.2.4. Oromotor/Verbal Subscale

All children, also those aged <6 months, presented vocalization/oral movement. By 24 months, all children reached the ceiling of the subscale, producing intelligible verbalizations “defined in the CRS-R as two different words comprising a consonant-vowel-consonant blend” [20] (p. 1643); the lowest age at which this behavior was consistently exhibited by all children was 18.90 months, while the youngest children displaying it were 18.01 months.

#### 3.2.5. Communication Subscale

No child < 13 months (29 of the total 64 children) exhibited non-functional intentional communication, “defined in the CRS-R as a clearly discernible verbal or gestural “yes” or “no” response to two of six visual or aurally based situational orientation questions or the modified CRS-P picture book set” [20] (p. 1643). A total of 8 children, all aged between 13 and 32 months, met the criteria for this item. Moreover, 27 of the total 64 children (42%) across the age range exhibited functional intentional communication (FC), “defined in the CRS-R as clearly discernible and accurate verbal or gestural “yes” or “no” responses to six consecutive visual or aurally based situational orientation questions or by the modified CRS-P criteria, which require clearly discernible and accurate verbal or gestural “yes” or “no” responses to six consecutive questions about images in a picture book” [20] (p. 1643). The lowest age at which this behavior was consistently exhibited by all children was 33.67 months. Only two children displayed FC before 33.67 months of age, who were aged 20.18 and 24.69 months, respectively.

#### 3.2.6. Arousal Subscale

Additionally, 33 of 64 children maintained continuous eye opening without stimulation across the entire examination. The youngest child who met the criterion for the Attention item (i.e., no more than three occasions where the child failed to respond to a verbal prompt) was 24 months of age; by 36 months, all children did. No child is required to be stimulated to maintain wakefulness and eye opening. For children aged <6 months, the examination was carried out at an appropriate time of day, away from the (assumed) time of rest, in order to optimize the observation of behavioral responses. Therefore, 100% of very young children (<6 months) exhibited eye opening without stimulation (Arousal subscale, 100%).

### 3.3. Items Indicating Emergence to Conscious State

In CRS-P [20], in accordance with CRS-R [26] and the actual diagnostic criteria, the emergence from DoC is indicated by the meeting of at least one of the following items: functional object use (FOU) of the Motor subscale and functional communication (FC) of the Communication subscale. All children by 36 months displayed FOU and FC. In detail, FOU was displayed by 46 of the total 64 children across the age range (72%), being displayed by all 8 children aged at least 18 months; the lowest age at which this behavior was consistently exhibited by all children was 14.43 months. In detail, such a behavior was displayed by 7 of 8 children aged 12–<18 months (87.5%), and 3 of 10 children aged 6–<12 months (30%). The youngest child who exhibited FOW was 7.46 months, while no child aged 0–<6 months did (0%). With respect to FC, in the whole sample, 27 of 64 children (42%) across the age range exhibited such a behavior; the lowest age at which this behavior was consistently exhibited by all children was 33.67 months. In detail, such a behavior was displayed by 2 of 6 children aged 24–<36 months (66.7%), and by 1 of 8 children aged 18–<24 months (12.5%). The youngest child who showed this behavior was 20.18 months old while no child aged <18 months did so. Thus, from a developmental perspective, FOU is the first criterion reached by typically developing children. All 14 children aged between 18 and 36 months displayed FOU, but only 5 (36%) showed FC. There were no cases exhibiting FC in the absence of FOU.

Table 3 depicts the number and percentage of children who met the criteria for FOU and FC.

## 4. Discussion

This study examined the performance of 64 Italian typically developing children aged 3 months to 5:7 years (68 months) on the Italian CRS-P [30] to obtain a preliminary validation of the tool, in accordance with the validation process of the original CRS-P for English-speaking countries [20]. However, compared to such a previous work that included children between the ages of 8 months and 4:9 years (59 months), this study documented behavioral responses in a wider age range, also comprising children aged <6 months. This allowed identifying the highest competences exhibited by very young children (<6 months) across the different subscales: localization to sound (Auditory scale; 90%), visual pursuit (Visual subscale; 50%), automatic motor response (Motor subscale, 40%), vocalization/oral movement (Oromotor/Verbal subscale, 100%), and eye opening without stimulation (Arousal subscale, 100%). None exhibited non-functional intentional communication (Communication subscale).

As in the English version of the CRS-P [20], a strong inter-rater reliability for both the subscale and total score was found, indicating a very high agreement between examiners. In addition, children’s performance was confirmed to correlate with age at administration: older children performed better. The correlation for ages was moderate for the Motor subscale and strong for the Auditory, Visual, Oromotor/Verbal, Communication, and Arousal subscales.

By 14 months of age, all children reached the ceiling in the Motor subscale and by 20 months in the Visual subscale. For subscales including language-mediated behaviors, the ceiling was reached by 19 months in the Oromotor/Verbal subscale, by 20 months in the Auditory subscale, and by 34 months in the Communication subscale. This confirmed the finding from the preliminary validation study of the original CRS-P [20] on the impact of language ability on children’s performance; it was also in line with developmental milestones of children with typical development reported in the extant literature, suggesting that motor and visual development occurs prior to language maturation [31]. Due to the later emergence of language-related abilities, our study corroborates the recommendation of Slomine and collaborators [20] to be cautious in employing command-following (Auditory subscale) in very young children with acquired brain injury.

With respect to behaviors indicating emergence to state of consciousness, findings reveal that all children aged at least 14 months displayed FOU. In addition, 87.5% of children aged 12–<18 months and 30% of those aged 6–<12 months did so. These data are similar to the results obtained in the American context [20], where FOU was consistently shown by 12 months. Even for the Italian context, FC proved to be a more challenging behavior, being consistently displayed by 34 months. The youngest child who exhibited it was aged 20 months. In the American sample, children had to be of at least 36 months of age to consistently exhibit FC.

The Italian CRS-P was able to discriminate between VS and MCS behaviors in the youngest children, with all children > 6 months demonstrating features of MCS across visual and motor abilities; indeed, in line with data from the American sample [20], fixation, visual pursuit, or object localization in the Visual subscale and automatic motor response in the Motor subscale were exhibited by this age. Even for children between 3 and 6 months of age, fixation or visual pursuit in the Visual subscale were consistently displayed; 90% of children (age range: 3.48–5.88 months) exhibited localization to noxious stimulation, object manipulation, or automatic motor response in the Motor subscale, with a unique child, the youngest of the sample of 3.25 months of age, displaying only flexion withdrawal. Therefore, the Visual and Motor subscales of the CRS-P allowed distinguishing between VS and MCS as early as 3 months of age. Differently, language-based behaviors of MCS were consistently displayed by older children: command-following (consistent movement to command, Auditory subscale) by 20 months, yes/no responses—FC—(Communication subscale) by 34 months, and intelligible verbalization (Oromotor/Verbal subscale) by 19 months. Compared to the American sample, in which these behaviors were consistently displayed by 45, 36, and 24 months, respectively, Italian children exhibited them earlier. These age differences warrant further exploration in a larger sample. Until then, caution should be used when comparing the distribution of DoC classifications between Italian-speaking and English-speaking young children assessed through the Italian and original CRS-P, respectively. A reliable comparison seems to be warranted only by considering the Oromotor/Verbal and Motor subscales.

However, regarding the Visual subscale, it is important to make a consideration about administration, based on our clinical experience. Indeed, between 5 and 12 months of age, 5 of the total 14 children spontaneously showed object localization (reaching), but did not meet the criteria for the previous item, i.e., visual pursuit (score = 3). This seems to have occurred because they demonstrated spontaneous visual pursuit in the presence of an object, especially if it was a toy or their mother’s face, but did not show such behavior when using a mirror, as required by the original CRS-P [20] instructions. Therefore, we suggest modifying the administration of item 3 of the Visual subscale by replacing the mirror with a child-friendly object; this would not alter the corresponding underlying neurological function, a fact that was indicated as an important rule to follow in the process of adapting the CRS-R to the CRS-P [20].

In some cases, behavioral problems had a negative impact on performance: some children exhibited oppositional behavior or distraction or refused to respond to commands during the assessment. To limit these issues, all children were assessed in the same setting in the presence of their caregiver(s) with the aim of maximizing interaction and favoring emotional well-being. The presence of family caregivers was found to improve the detection of cortically mediated behaviors even in adults with DoC [32], leading to higher scores on neurobehavioral scales [33,34]. Obviously, at a very young age, caregiver involvement is critical to allow children to exhibit more complex behaviors, with the same potential benefits observed in adults in improving diagnostic accuracy. In addition, CRS-P examiners should have a robust knowledge on developmental variables that influence motor and cognitive performance as well as on DoC assessment to interpret performance and distinguish features of the distinct stages of DoC.

In relation to clinical implications, this study allowed the identification of the behavioral repertoire of typically developing children with respect to behaviors recognized as being able to discriminate levels of consciousness, which constitutes the basis for the identification of any deviations from the normal growth curve in children with neurological condition. Our work also demonstrated that some scales of the Italian CRS-P (the Visual and Motor subscales) are able to distinguish between VS and MCS as early as 3 months of age; in addition, as all children aged ≥14 months met the criteria for FOU, the CRS-P may be considered appropriate for detecting one of the two criteria indicating emergence to consciousness in children of this age. However, as typically developing children reached the ceiling of the CRS-P by 34 months, caution should be considered when adopting the scale at a younger age. Nevertheless, compared to other tools previously tested with children with DoC [13,22,24,25], such as LOCFAS [28,29] or CRS-R [26], which were shown to be inapplicable in children younger than 4 and 5 years of age, respectively, CRS-P seems to have the advantage to be usable at a younger age. Given that the literature on DoC in pediatrics is mainly observational and clinical details are often inconsistently described [10], this study may provide important knowledge to inform future clinical practice and research directions. Finally, given the subjective variability in the attainment of developmental milestones, we stress the importance of collecting the accurate developmental history data on children with DoC prior to the administration of neurobehavioral scales.

Future research is needed to further test the use of the CRS-P in Italy and the United States with typically developing young children to better understand the reasons for age discrepancies between the two countries in some cognitive and motor skills. Next, studies should test and report data on the administration of the CRS-P in children with DoC and explore the agreement between this tool and other scales to identify any discrepancies and capture the strengths and weaknesses of each. Although several standardized neurobehavioral assessment tools have been used so far with children to diagnose states of DoC, data on reliability and validity are still lacking [9]. Additionally, especially for the youngest children, a multimodal assessment should be preferred when visual and motor skills are impaired [9]. However, the tools included in such assessment should be previously tested and validated by ad hoc studies to inform practice guidelines.

Another important step for research in the field should be the identification of behaviors, so far not included in clinical guidelines, that represent new potential signs of consciousness. This could help to discriminate levels of consciousness more easily.

The limitations of the study should be acknowledged. First, a limited sample consisting of children from families who volunteered was included, which limits the generalizability of the results. Second, as in the original preliminary validation study [20], the full complement of CRS-P command and yes/no question sets were adopted, which could have improved scores in some cases. In addition, this administration procedure could partially differ from the one used in clinical practice, possibly further limiting generalization. Finally, comparison with other neurobehavioral scales used to assess children with DoC was not possible, as no other tool was administered.

## 5. Conclusions

Children emerging from DoC often have neurological deficits that alter motor and cognitive functions and may experience the loss of previously achieved developmental milestones. This may lead to developmental delays of several months compared with typical development. This study indicated that typically developing children are able to meet the criteria for all items of the Italian CRS-P by the age of 34 months, which suggests caution in adopting the scale at a younger age. However, considering the behaviors examined by the various subscales, CRS-P was shown to capture the transition from VS to MCS at a very young age, as early as 3 months of age in the Visual and Motor subscales.

Instead, modifications to the language-mediated items that discriminate between VS and MCS in the Oromotor/Verbal, Auditory, and Communication subscales are recommended to better capture the different characteristics of the stages of DoC in children younger than 19, 20, and 34 months of age, respectively. The transition to eMCS was consistently detected by 14 months of age, when functional object use was shown. However, in children with DoC, this behavior is often hindered by the child’s emerging motor abilities, which may postpone the age of stable skill acquisition.

Globally, findings suggest that the assessment of children aged <34 months should specifically take into account the different ages related to the achievement of developmental milestones in the various subscales and, in children with DoC, the possible interfering neurological deficits caused by the acquired brain injury.

For this reason, examinations should be performed by specialists trained in such assessments and with adequate knowledge of child development and DoC characteristics. Future studies are needed to define new modifications to the scale to improve its diagnostic accuracy. For example, our clinical experience suggests the usefulness of modifying the administration procedure of item 3 of the Visual subscale, which assesses visual pursuit, by replacing the mirror with a child-friendly object or the mother’s face, since children may exhibit this behavior only in the presence of such stimuli.

## Figures and Tables

**Table 1:** Inter-rater reliability of CRS-P subscale and total scores.

	Psychologist 1		Psychologist 2		ρ_c_
Subscales	Median [Range]	Mean (SD)	Median [Range]	Mean (SD)	
Auditory	4.00 [1.00, 4.00]	3.14 (0.98)	4.00 [1.00, 4.00]	3.14 (0.98)	1
Visual	5.00 [2.00, 5.00]	4.16 (1.16)	5.00 [2.00, 5.00]	4.16 (1.16)	1
Motor	6.00 [3.00, 6.00]	5.64 (0.69)	6.00 [3.00, 6.00]	5.66 (0.64)	0.97
Oromotor/Verbal	3.00 [2.00, 3.00]	2.55 (0.50)	3.00 [2.00, 3.00]	2.55 (0.50)	1
Communication	1.00 [0.00, 2.00]	1.00 (0.96)	1.00 [0.00, 2.00]	1.00 (0.96)	1
Arousal	2.00 [2.00, 3.00]	2.43 (0.50)	2.00 [2.00, 3.00]	2.41 (0.50)	0.95
Total Score	20.50 [11.00, 23.00]	18.91 (4.32)	20.50 [11.00, 23.00]	18.91 (4.27)	1

ρ_c_ = Lin’s concordance correlation coefficient.

**Table 2:** CRS-P: descriptive statistics for subscale and total scores by age group.

CRS-P Subscales								
Mean, Median (SD)								
								Total Score
Age Group (Months)	n	Auditory	Visual	Motor	Oromotor/Verbal	Communication	Arousal	Mean, Median (SD), and Range
0–<6	10	1.90, 2 (0.32)	2.70, 3 (0.78)	4.10, 4 (0.99)	2.00, 2 (0.00)	0.00, 0 (0.00)	2.00, 2 (0.00)	12.70, 13 (1.50) 10–15
6–<12	10	2.00, 2 (0.00)	3.60, 4 (0.52)	5.30, 5 (0.48)	2.00, 2 (0.00)	0.00, 0 (0.00)	2.00, 2 (0.00)	14.90, 15 (0.57) 14–16
12–<18	8	2.50, 2 (0.76)	4.13, 4 (0.64)	5.88, 6 (0.35)	2.00, 2 (0.00)	0.13, 0 (0.35)	2.00, 2 (0.00)	16.63, 16.5 (1.30) 15–18
18–<24	8	3.50, 3.5 (0.54)	4.88, 5 (0.35)	6.00, 6 (0.00)	2.75, 3 (0.46)	0.88, 1 (0.64)	2.00, 2 (0.00)	20.00, 20 (1.31) 18–22
24–<36	6	4.00, 4 (0.00)	5.00, 5 (0.00)	6.00, 6 (0.00)	3.00, 3 (0.00)	1.67, 2 (0.52)	2.67, 3 (0.52)	22.33, 23 (1.03) 21–23
36–<48	9	4.00, 4 (0.00)	5.00, 5 (0.00)	6.00, 6 (0.00)	3.00, 3 (0.00)	2.00, 2 (0.00)	3.00, 3 (0.00)	23.00, 23 (0.00) 23–23
48–<60	7	4.0, 4 (0.00)	5.00, 5 (0.00)	6.00, 6 (0.00)	3.00, 3 (0.00)	2.00, 2 (0.00)	3.00, 3 (0.00)	23.0, 23 (0.00) 23–23
60+	6	4.00, 4 (0.00)	5.00, 5 (0.00)	6.00, 6 (0.00)	3.00, 3 (0.00)	2.00, 2 (0.00)	3.00, 3 (0.00)	23.00, 23 (0.00) 23–23
Total Range	64	3.11, 4 (0.98) 1–4	4.30, 5 (0.95) 2–5	5.58, 6 (0.81) 2–6	2.53, 3 (0.50) 2–3	0.97, 1 (0.94) 0–2	2.41, 2 (0.50) 2–3	18.89, 20 (4.16) 10–23

Note: Possible ranges for subscale and total scores: Auditory (0–4), Visual (0–5), Motor (0–6), Oromotor/Verbal (0–3), Communication (0–2), Arousal (0–3), Total (0–23).

**Table 3:** Number and percentage of children who met criteria for functional object use (FOU) and functional communication (FC) by age range.

	FOU		FC	
Age Group (Months)	n	%	N	%
0–<6	0/10	0%	0/10	0%
6–<12	3/10	30%	0/10	0%
12–<18	7/8	87.5%	0/8	0%
18–<24	8/8	100%	1/8	12.5%
24–<36	6/6	100%	4/6	66.7%
36–<48	9/9	100%	9/9	100%
48–<60	7/7	100%	7/7	100%
60+	6/6	100%	6/6	100%

Note. FC = functional communication; FOU = functional object use.

## Data Availability

The data presented in this study are available on request from the corresponding author due to privacy restrictions.
